# Temporary hemiepiphysiodesis using an eight‐plate implant for coronal angular deformity around the knee in children aged less than 10 years: efficacy, complications, occurrence of rebound and risk factors

**DOI:** 10.1186/s12891-020-03915-w

**Published:** 2021-01-09

**Authors:** Zhen-Zhen Dai, Zhen-Peng Liang, Hao Li, Jing Ding, Zhen-Kai Wu, Zi-Ming Zhang, Hai Li

**Affiliations:** Department of Pediatric Orthopedics, Xin Hua Hospital, Shanghai Jiao Tong University School of Medicine, 1665 Kongjiang Road, Yangpu District, 200092 Shanghai, China

**Keywords:** Coronal angular deformity of the knee joint, Temporary hemiepiphysiodesis, Eight-Plate, Paediatric

## Abstract

**Background:**

Temporary hemiepiphysiodesis (TH) using an eight-Plate implant is one of the most common surgeries used for the correction of coronal angular deformities around the knee in adolescents. However, few studies have focused on children aged less than 10 years treated with TH using an eight-Plate implant. The purpose of this study was to investigate the efficacy, correction velocity, and complications of TH with an eight-Plate implant as well as the occurrence of rebound and risk factors in this population.

**Methods:**

This retrospective study included a total of 135 physes (101 knees) from 66 children (mean age of 4.69 years old, range from 1 to 10 years old) who underwent TH with an eight-Plate implant to correct coronal genu angular deformities in our hospital. Related clinical factors were recorded and analysed by multivariable linear and logistic regression models.

**Results:**

The mean deformity correction period was 13.26 months, and the mean follow-up after eight-Plate removal was 12.71 months. In all, 94.06% (95/101 knees) of the genu angular deformities were completely corrected. Non-idiopathic genu angular deformity was found to be an independent risk factor for deformity correction failure (odds ratio (OR) = 2.47). The femoral correction velocity was significantly higher than the tibial correction velocity (1.28° vs. 0.83° per month, *p* < 0.001). After adjustment for other factors, younger children had higher correction velocities in the distal femur; however, genu valgum and idiopathic deformities were associated with higher correction velocities in the proximal tibia. In addition, we found three (3/101, 2.97%) knees with genu valgum that experienced rebound after removal of the eight-Plate, while five (5/101, 4.95%) knees with non-idiopathic genu angular deformity experienced screw loosening. No other complications were found, and non-idiopathic deformity was the only risk factor for complications (OR = 3.96). No risk factor was found for rebound in our study.

**Conclusions:**

TH using an eight-Plate implant is an effective procedure for coronal genu angular deformities with a low incidence of complications and rebound in patients younger than 10 years old. For this population, TH using an eight-Plate should be considered as soon as the deformity stops responding to conservative treatments. The parents of children younger than 10 years of age with non-idiopathic deformities should be informed preoperatively that the deformity may be prone to correction failure or screw loosening after eight-Plate implantation.

## Background

Coronal angular deformity around the knee is a common phenomenon with multiple aetiologies (idiopathic, posttraumatic, rickets, etc.) in children and can cause abnormal gait function and affect the surrounding joints in the process of growth [1–3]. When pathologic coronal angular deformity around the knee does not resolve spontaneously, or even worsens, surgical intervention is often indicated [3–5]. Temporary hemiepiphysiodesis (TH) has gradually become a widely used procedure for these patients [1, 6, 7]. At present, the eight-Plate implant suggested by Stevens [8] is the most popular implant used for TH surgery because of the simplicity of the operation, the safety of the implant and the low rate of complications [1, 9]. However, most studies advocate delaying TH until 8–10 years of age due to concerns about permanent physeal damage or the occurrence of rebound [1]. Few studies have focused on genu angular deformity in patients under 10 years of age [10, 11]. There is only one study about genu angular deformity in which the age of the patients was under 10 years old. However, the sample size was relatively small (24 patients), and the study mainly focused on the effectiveness of TH using an eight-Plate [11]. Therefore, factors influencing the effectiveness, occurrence of rebound and complications of TH using eight-Plate implants for patients under 10 years of age remain unclear.

The primary purpose of the present research was to evaluate the efficacy, correction velocity, complications and occurrence of rebound of TH using eight-Plate implants for these young patients and to investigate the possible risk factors.

## Methods

After institutional review board (IRB) approval, we retrospectively reviewed all of the patients with coronal angular deformities around the knee who were treated with TH procedures using an eight-Plate in the Department of Paediatric Orthopaedics in our hospital from January 2014 to December 2017.

The inclusion criteria were as follows: (1) patients younger than 10 years old with open physis confirmed by X-ray, (2) patients diagnosed with genu coronal angular deformities by examination of lower limb mechanical alignment in full-length lower extremity standing anteroposterior radiographs regardless of the causes, (3) patients treated with TH surgery using a single eight-Plate per physis around the knee, and (4) patients with complete medical data and follow-up for more than 12 months following eight-Plate removal.

The exclusion criteria were as follows: (1) history of osteotomy or any other surgeries before the TH procedure and (2) bone bridge formation in the physis following growth plate injury.

In our hospital, for children with coronal angular deformity around the knee who have no improvement after one year of observation, TH using an eight-Plate is routinely planned. After the preoperative clinical and radiographic evaluations were finished, the TH procedures were accomplished according to the methods of Stevens [8] by multiple senior paediatric orthopaedists as follows: under fluoroscopic guidance, two 1.5-mm guide K-wires were introduced, parallel to the physis, into the metaphyseal and epiphyseal regions, and then a 2-cm incision was made between the two guide K-wires. The eight-Plates (length: 25 or 30 mm, thickness: 1 mm) were placed outside the periosteum along the guide pins, and fully threaded self-tapping cannulated screws (diameter: 3.0 or 4.5 mm, length being approximately half of the width of the physis) were implanted along the guide pins. After confirming the position of the plate and screws by fluoroscopy, we released the soft tissue compressed by the plate to ensure that the joint moved freely.

**Fig. 1:**
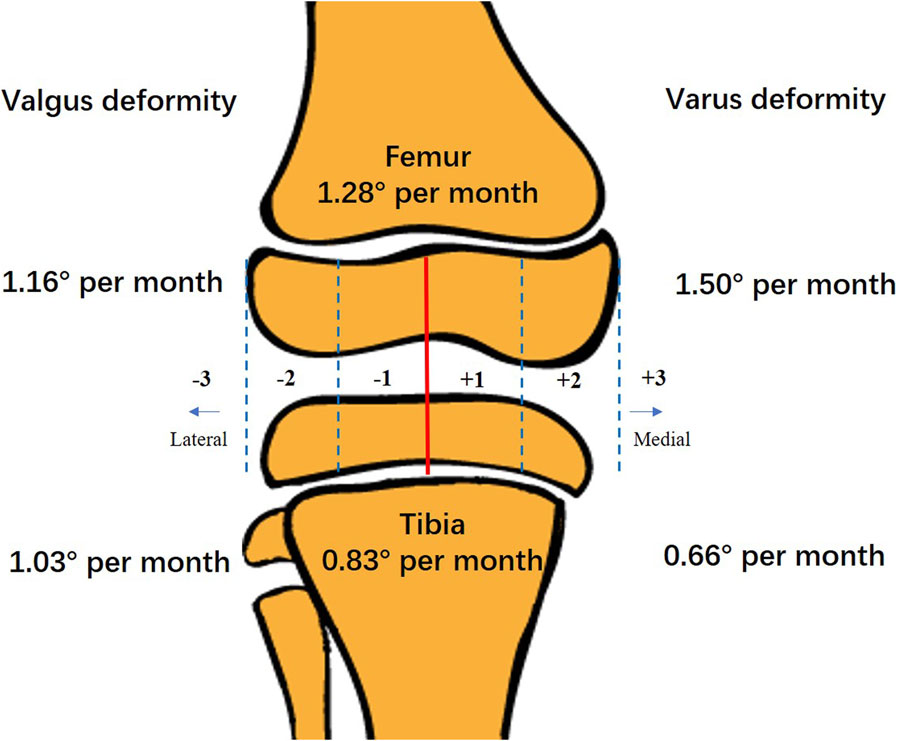
The zones of the mechanical axis [10] and sketches of the correction velocities of the femur and tibia in different genu angular deformities. The mechanical axis of the lower extremities passing through zone 1 represents normal alignment and includes the middle 2 quadrants (medial: +1; lateral: -1). The mechanical axis of the lower extremities passing through zones − 2 and − 3 represents genu valgum deformity and through + 2 and + 3 represents genu varum deformity. The overall femoral correction velocity (1.28° per month) was significantly faster than the tibial correction velocity (0.83° per month) (*P* = 0.000). For the femur, the correction velocity for a varus deformity was significantly faster than that for a valgus deformity (1.50° vs. 1.16° per month, *P* = 0.033). For the tibia, the correction velocity for a valgus deformity was significantly faster than that for a varus deformity (1.03° vs. 0.66° per month, *P* = 0.010)

Postoperatively, instead of immobilization, early weight-bearing and physical activity without any limitation were required at one and two weeks, respectively. All patients received regular follow-up every three months. We used 88° and 87° as the normal values for the mechanical lateral distal femoral angle (mLDFA) and the mechanical medial proximal tibial angle (mMPTA), respectively, according to the previous literature [12]. Once neutralization of the mechanical axis to within zone − 1 to 1 (Fig. 1) or overcorrection of the mLDFA or mMPTA within 5 degrees was achieved, the plates and screws were removed [7, 10, 11]. Patients did not return to full activity until complete surgical wound healing was achieved (approximately 2 weeks).

We collected clinical data, including demographic, radiographic and outcome data. All radiographic indexes were obtained and measured by two senior physicians in PACS (Picture Archiving and Communication System, Uni-Wed, 6.1, EBM Technologies, Shanghai), including the mLDFA, mMPTA, screw divergence angle (SDA), screw lengths, coronal width of the epiphysis and the location of the centre of the mechanical axis (zones 1, 2, 3, -1, -2 and − 3) [10].

Complications, such as screw loosening, screw breakage, wound infection, premature physis and limited range of movement, were collected. Based on previous studies [12–14], we considered anatomical angle changes > 3 degrees (mLDFA 88° or mMPTA 87°) after eight-Plate removal to be indicative of the rebound phenomenon, and close observation, revision hemiepiphysiodesis or osteotomy was performed as necessary.

### Statistical analysis

The correlation of these clinical factors was assessed by Fisher’s exact test or the Wilcoxon test for categorical variables and by t-test or analysis of variance (ANOVA) for continuous variables. Risk factors for correction velocity were evaluated by multivariate linear regression models, and the adjusted coefficients with their 95% confidence intervals (CIs) were also obtained. Risk factors for experiencing rebound were evaluated by multivariate logistic regression models, and odds ratios (ORs) with their 95% CIs were obtained. Correlation significance was assessed by Pearson’s correlation test. To model the change in SDA and the correction angle of the studied limb, a linear regression analysis (least-squares method) was performed. Factors included in the multivariate model were those with *p* < 0.5 in the univariable analysis or those found to be significant predictive factors in the literature. Statistical tests were considered significant at *p* < 0.05. All analyses were performed with the statistical software Stata/SE for Windows (version 15.0; StataCorp LLC, College Station, TX, USA).

## Results

**Fig. 2:**
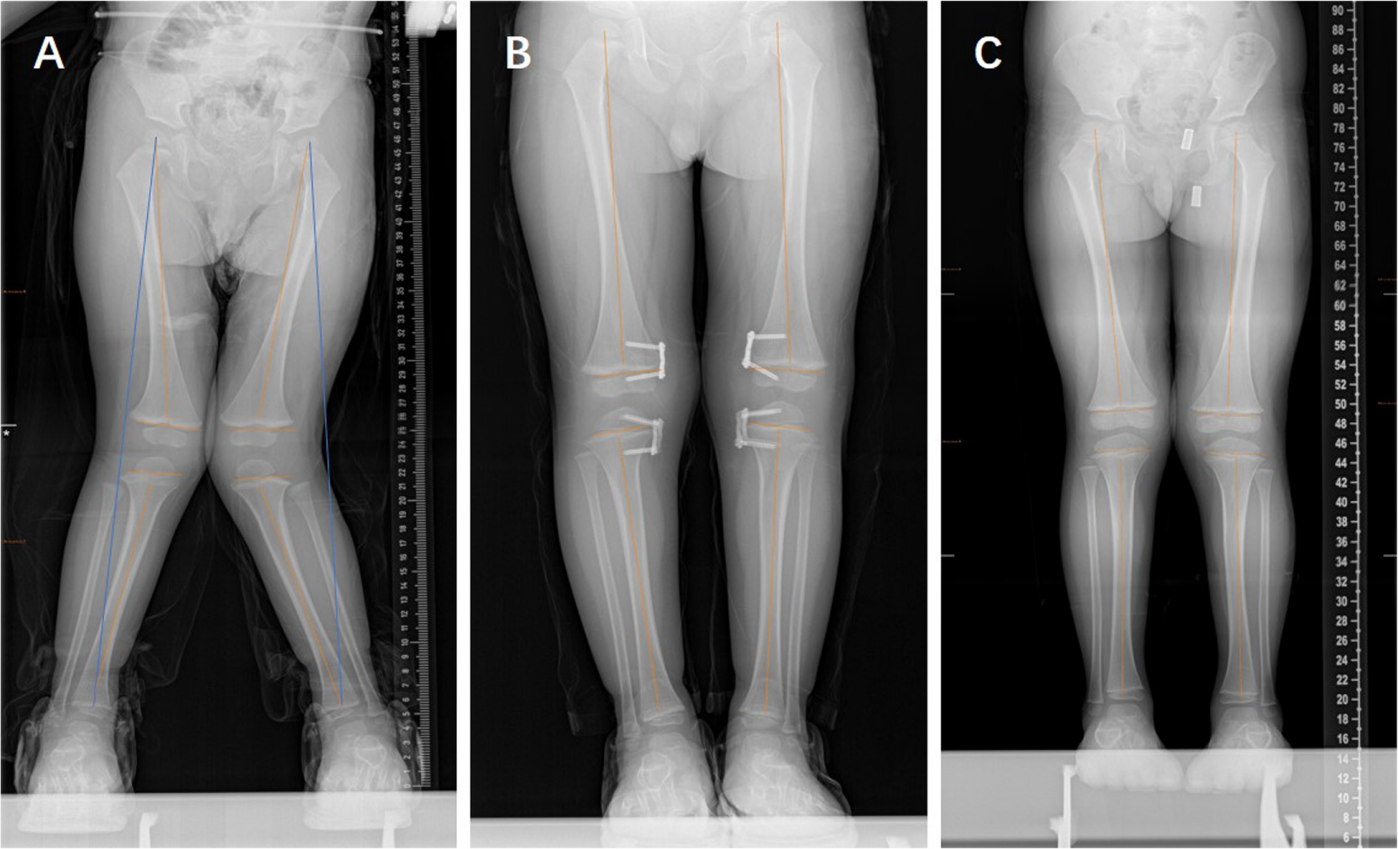
Comparison of full-length anteroposterior standing radiographs preoperatively, before removing the inner fixation device and at the last follow-up in a 2-year-old boy with bilateral idiopathic genu valgus deformities. **a**, pre-operation; **b**, before removing the inner fixation device (6 months TH surgery); **c**, the last follow-up (22 months after removing the inner fixation device)

**Fig. 3:**
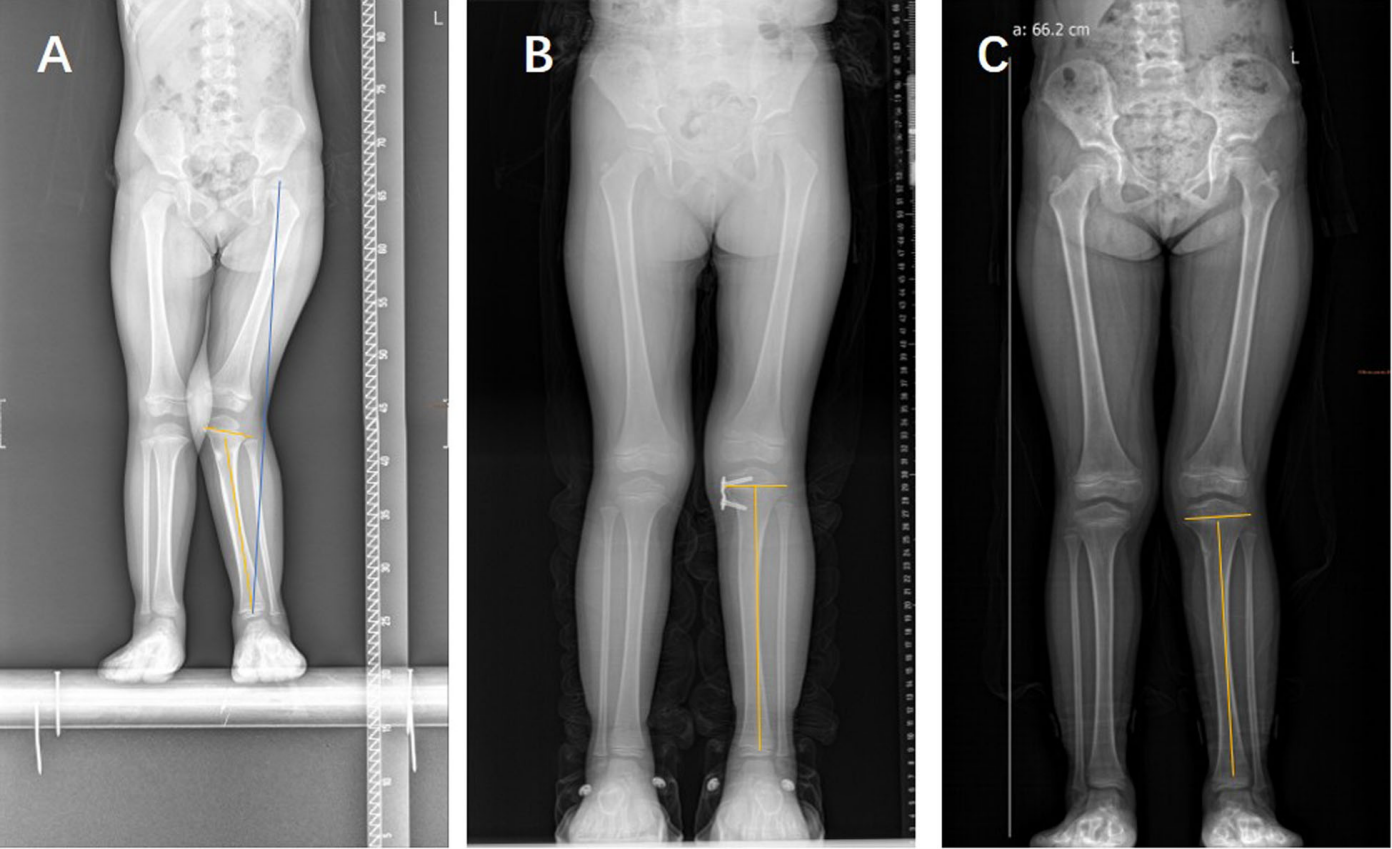
Comparison of full-length anteroposterior standing radiographs preoperatively, before removing the inner fixation device and at the last follow-up in a 3-year-old boy with unilateral posttraumatic genu valgus deformity. **a**, pre-operation; **b**, before removing the inner fixation device (18 months after TH surgery); **c**, the last follow-up (24 months after removing the inner fixation device)

A total of 66 patients with 135 physes (101 knees) were included in our study (Table 1): 40 boys and 26 girls. The mean age at the time of surgery was 4.69 years old, ranging from 1 to 10 years old. Idiopathic coronal angular deformity of the knee joint was the most common deformity (*n* = 28, 42.42%) (case shown in Fig. 2), followed by posttraumatic deformity (*n* = 14, 21.21%) (case shown in Fig. 3). In all, 66.67% (44/66) of patients had genu valgum, whereas 33.33% (22/66) of patients had genu varum. Thirty percent (20/66) of patients had plates inserted into both the distal femur and the proximal tibia. The mean deformity correction time was 13.26 months (range from 4 to 40 months), and the mean follow-up after eight-Plate removal was 12.71 months (range from 12 to 24 months) (Table 1). The average mLDFA correction was 13.38° (range from 2.6 to 32.7°), and the average mMPTA correction was 10.05° (range from 0.45 to 22.21°).

**Table 1:** Demographics, treatment characteristics and outcomes

Variable	Study cohort (*n* = 66)
Age* (yrs) [range]	4.69 ± 2.59 [1,2,3,4,5,6,7,8,9,10]
Sex (no. [%])	
Male	40 (60.61%)
Female	26 (39.39%)
Weight* (kg) [range]	21.28 ± 7.59 [9.3–46]
Unilateral	32 (48.48%)
Aetiology (no. [%])	
Idiopathic	28 (42.42%)
Posttraumatic	14 (21.21%)
Rickets	9 (13.64%)
Metaphyseal dysplasia	6 (9.09%)
Osteochondroma	5 (7.58%)
Infection	4 (6.06%)
Genu valgum (no. [%])	44 (66.67%)
Eight-Plate implantation location (no. [%])	
Distal femur alone	24 (36.36%)
Proximal tibia alone	22 (33.33%)
Both sites	20 (30.30%)
Correction success rate (no. [%]) ^**a**^	95/101 (94.06%)
Distal femur alone	39/41 (95.12%)
Proximal tibia alone	23/26 (88.46%)
Both sites	33/34 (97.06%)
Complication (no. [%]) ^**a**^	5/101 (4.95%)
Rebound phenomenon (no. [%]) ^**a**^	3/101 (2.97%)
Deformity correction time* (months) [range]	13.26 ± 7.73 (4–40)
Follow-up after eight-Plate removal* (months) [range]	12.71 ± 1.98 (12–24)

Values are presented as the mean ± SD (range) or frequency (percentage)

^a^calculated according to the number of limbs

### Correction velocity and influencing factors

The overall femoral correction velocity (1.28° per month) was significantly higher than the tibial correction velocity (0.83° per month) (*p* < 0.001). For the femur, the correction velocity for varum deformities was significantly higher than that for valgum deformities (Fig. 1 1.50° vs. 1.16° per month, *P* = 0.033). However, for the tibia, the correction velocity for valgum deformities was significantly higher than that for varum deformities (Fig. 1 1.03° vs. 0.66° per month, *P* = 0.010).

The mean preoperative SDA was 7.27°, ranging from − 5.6° to 33.3°. The screw length was 45.44% of the coronal width of the femoral physis, ranging from 28.51 to 61.18%. However, neither was associated with the correction velocity (Table 2).

**Table 2:** Analysis of correction velocity and influencing factors by multiple linear regression

	Crude Coefficient (95% CI^a^)	*P*	Adjusted Coefficient (95% CI)	*P*
**Correction Velocity in mLDFA**				
Age	-0.12 (-0.18- -0.07)	**< 0.001**	-0.17 (-0.26- -0.07)	**0.001**
Weight	-0.03 (-0.05- -0.01)	**0.013**	0.02 (-0.01-0.05)	0.219
Unilateral	0.16 (-0.26-0.58)	0.453	-0.15 (-0.59-0.29)	0.496
Genu angular deformity type	0.34 (0.03–0.64)	**0.033**	0.31 (-0.05-0.67)	0.089
Preoperative SDA	-0.01 (-0.03-0.01)	0.353	-0.00 (-0.02-0.02)	0.799
Percent of screw length/coronal width of femoral physis	-0.01 (-0.03-0.02)	0.632	-0.01 (-0.03-0.02)	0.517
Aetiology	-0.08 (-0.38-0.23)	0.622	0.05 (-0.29-0.39)	0.782
**Correction Velocity in mMPTA**				
Age	-0.09 (-0.14- -0.05)	**< 0.001**	-0.04 (-0.10-0.02)	0.201
Weight	-0.02 (-0.04- -0.00)	**0.015**	-0.01 (-0.03-0.01)	0.367
Unilateral	-0.04 (-0.35-0.26)	0.782	0.08 (-0.17-0.33)	0.504
Genu angular deformity type	-0.36 (-0.63- -0.10)	**0.010**	-0.41 (-0.65- -0.16)	**0.002**
Preoperative SDA	0.00 (-0.02-0.03)	0.711	-0.00 (-0.02-0.02)	0.993
Percent of screw length/coronal width of tibial physis	0.02 (-0.00-0.05)	0.056	0.02 (-0.00-0.04)	0.052
Aetiology	-0.60 (-0.86- -0.33)	**< 0.001**	-0.56 (-0.80- -0.33)	**< 0.001**

^a^CI indicates confidence interval

Bold values are statistically significant, *P* < 0.05

*mLDFA *mechanical lateral distal femoral angle, *mMPTA *mechanical medial proximal tibial angle, *SDA *screw divergence angle

Genu angular deformity type: Genu valgus vs. genu varus

Aetiology: Idiopathic vs. non-idiopathic

For the mLDFA correction velocity, age, weight and genu varum were found to be significant influencing factors in the univariate analysis (Table 2). After adjusting for other factors, the femoral correction velocity decreased with age (adjusted coefficient, -0.17; 95% CI, -0.26- -0.07, *p* = 0.001) (Table 2).

For the mMPTA correction velocity, age, weight, genu valgum and idiopathic aetiology were found to be significant influencing factors in the univariate analysis (Table 2). After adjusting for other factors, it was found that the tibial correction velocity was slower in genu varum (adjusted coef., -0.41; 95% CI, -0.65- -0.16, *p* = 0.002) and non-idiopathic genu angular deformities (adjusted coef., -0.56; 95% CI, -0.80- -0.33, *p* = 0.000) (Table 2).

### Relationship between changes in the Screw Divergence Angle (△SDA) and the correction angle

**Fig. 4:**
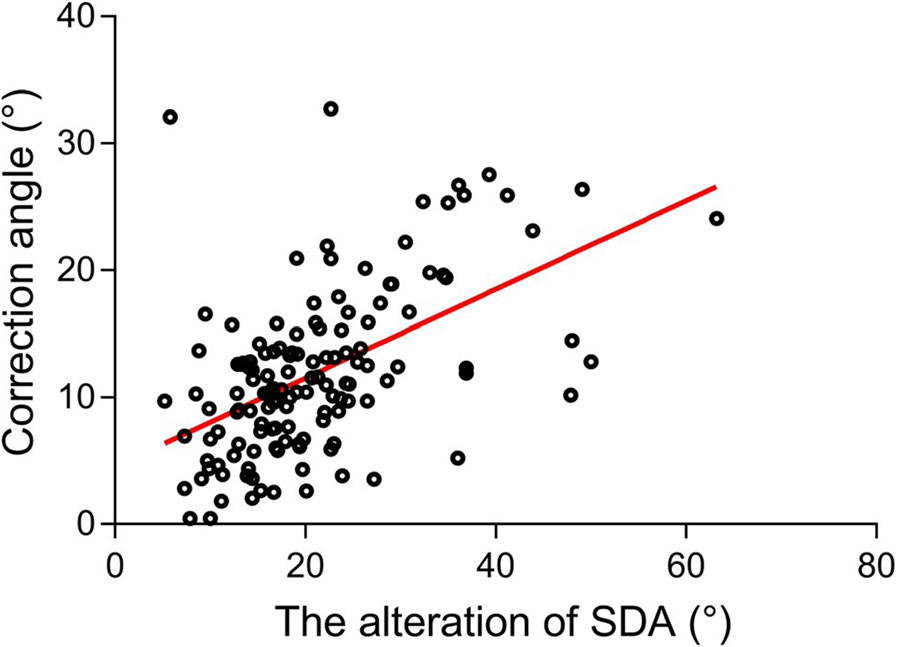
The scatter plot shows the relationship between the changes in SDA and the correction angle (*r* = 0.53, *P* < 0.0001). The fitted line was used to model the change in SDA and the correction angle (correction angle = 0.35*△SDA + 4.55 with coefficient of determination (R2) = 0.28) in linear regression analysis (least-squares method)

The mean △SDA was 21.07°, ranging from 5.2° to 63.2°, and it had a significant correlation with the mean correction angle (mean: 11.90°, range: 2.6° -32.7°) (*r* = 0.53, 95% CI: 0.39–0.64, *P* < 0.0001). For every one degree change in screw divergence, the correction angle changed 0.35 degrees (correction angle = 0.35*△SDA + 4.55, coefficient of determination (R2) = 0.28, Fig. 4).

### Deformity correction failure and risk factors

**Fig. 5:**
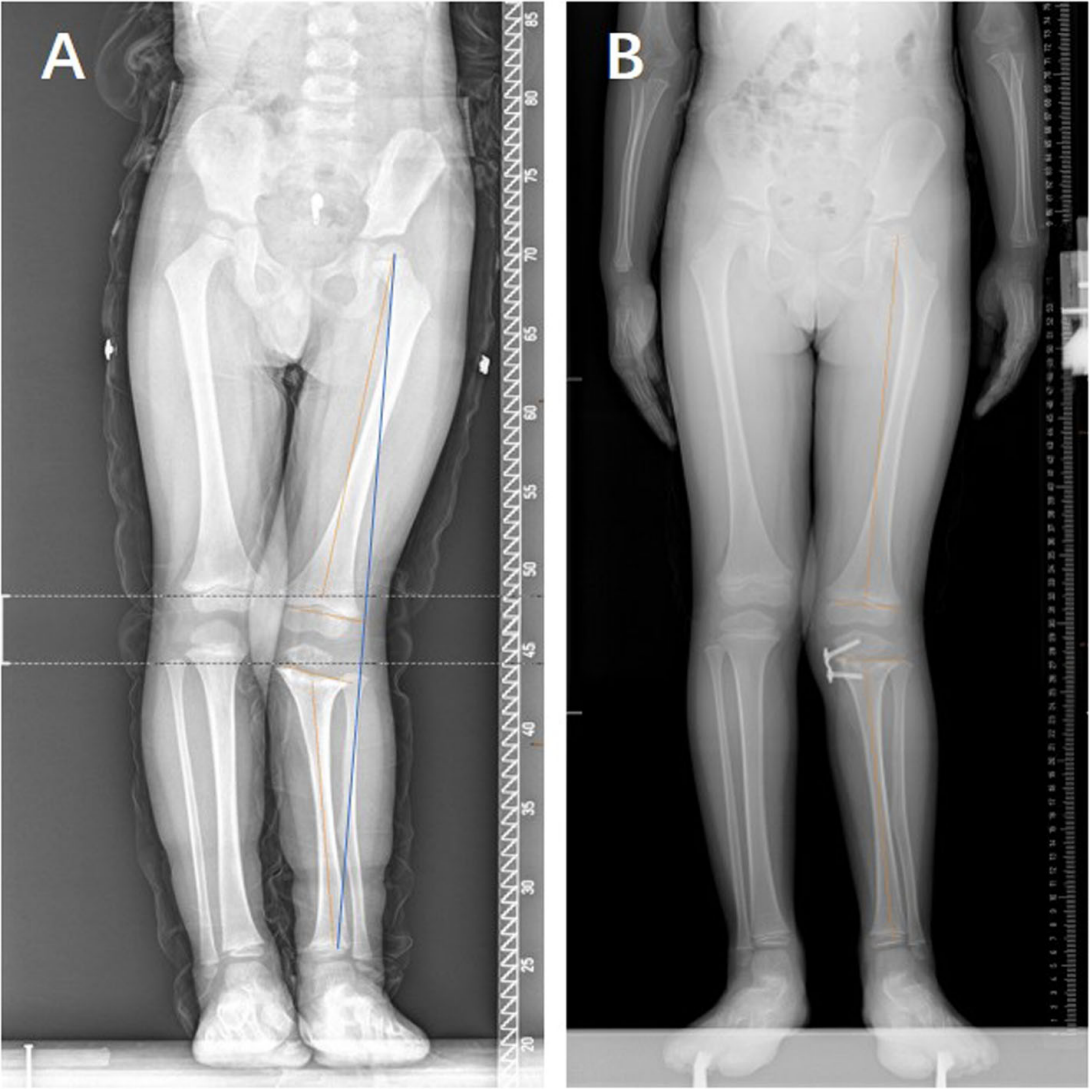
**a-b**, Deformity correction failure in a 3-year-old patient with unilateral genu angular deformity. **a**, pre-operation; **b**, poor treatment effect (20 months after TH surgery)

In all, six (6/101, 5.94%) patients experienced deformity correction failure (case shown in Fig. 5). Four patients with deformity correction failure underwent another TH surgery using an eight-Plate, and the other two patients underwent revision osteotomy. Five (5/6, 83.33%) patients with deformity correction failure had non-idiopathic aetiologies: rickets (*n* = 2), metaphyseal dysplasia (*n* = 2), and infection (*n* = 1). By multivariate analysis, non-idiopathic genu angular deformity was found to be an independent risk factor for deformity correction failure (OR = 2.47, 95% CI: 1.20–5.10, Table 3).

**Table 3:** Risk factors for correction failure by univariate analysis and logistic regression analyses

Influencing factors	Correction failure (*N* = 6)	Correction success (*N* = 95)	*P*	Logistic regression Odds ratio (95% CI^a^)	*P*
**Age**	5.75 ± 3.55 (1.5–10)	4.64 ± 2.55 (1–10)	0.481	1.46 (0.68–3.14)	0.331
**Weight**	21.10 ± 11.10 (9.6–35)	21.24 ± 7.26 (9.3–46)	0.977	0.96 (0.75–1.23)	0.762
**Unilateral**	1 (17)	31 (33)	0.662	5.23 (0.35–77.54)	0.229
**Genu valgus**	3 (50)	60 (63)	0.670	1.09 (0.11–11.06)	0.942
**Idiopathic**	1 (17)	47 (49)	0.056	2.47 (1.20–5.10)	**0.014**
**Correction location**			0.432	0.41 (0.10–1.77)	0.232
Distal femur alone	2 (33)	39 (41)			
Proximal tibia alone	3 (50)	23 (24)			
Both sites	1 (17)	33 (35)			
**Preoperative location of centre of the mechanical axis**^**b**^			0.211	7.65 (0.55-106.61)	0.130
2	1 (17)	46 (48)			
3	5 (83)	49 (52)			

^a^CI indicates confidence interval

^b^Preoperative location of the centre of the knee mechanical axis: zones 2, 3, -2 and − 3

Bold values are statistically significant, *P* < 0.05

Values are presented as the mean ± SD (range) or frequency (percentage)

### Rebound phenomenon and risk factors

Three patients (3/101 knees, 2.97%) with genu valgum (2 with it in the medial femur and 1 with it in the medial tibia) experienced rebound after removal of the eight-Plate. However, we found no risk factors by univariate or multivariate analysis (Table 4).

**Table 4:** Risk factors for rebound by univariate analysis and logistic regression analyses

Influencing factors	Rebound (*N* = 3)	Non-rebound (*N* = 98)	*P*	Logistic regression Odds ratio (95% CI^a^)	*P*
**Age**	6.67 ± 2.52	4.64 ± 2.60	0.297	2.03 (0.89–4.61)	0.092
**Weight**	22.17 ± 6.29	21.20 ± 7.52	0.817	0.83 (0.62–1.12)	0.231
**Unilateral**	2 (67)	30 (31)	0.235	0.18 (0.01–3.98)	0.279
**Genu valgus**	3 (100)	60 (61)	0.289		
**Idiopathic**	1 (33)	47 (48)	1.000	0.83 (0.04–16.52)	0.902
**Screw location**			0.518	0.45 (0.06–3.24)	0.430
Distal femur alone	2 (67)	39 (40)			
Proximal tibia alone	0 (0)	26 (26)			
Both sites	1 (33)	33 (34)			
**Location of the centre of the mechanical axis preoperatively**^**b**^			0.596	0.93 (0.05–16.23)	0.962
2	2 (67)	45 (46)			
3	1 (33)	53 (54)			

^a^CI indicates confidence interval

^b^absolute value of the location of the centre of the knee mechanical axis preoperatively (zones 1, 2, 3, -1, -2 and -3)

Values are presented as the mean±SD (range) or frequency (percentage)

Bold values are statistically significant, *P* < 0.05

### Complications and risk factors

Screw loosening was found in five patients (5/101 knees, 4.95%) with non-idiopathic genu angular deformity: rickets (*n* = 2), metaphyseal dysplasia (*n* = 1), and infection (*n* = 2). Non-idiopathic genu angular deformity was found to be an independent risk factor for screw loosening (OR = 3.96, 95% CI: 1.27–12.39, Table 5). No other complications, such as screw breakage, infection, physis preclosure or limited range of movement, were found in the follow-up.

**Table 5:** Risk factors for complications by univariate analysis and logistic regression analyses

Influence factors	Complications (*N* = 5)	No complications (*N* = 96)	*P*	Logistic regression Odds ratio (95% CI^a^)	*P*
**Age**	4.71 ± 2.61 (1.5–10)	4.64 ± 2.91 (1–8)	0.963	1.10 (0.48–2.55)	0.817
**Weight**	18.54 ± 3.10 (11.4–29)	21.37 ± 7.50 (9.3–46)	0.421	0.94 (0.71–1.23)	0.641
**Genu valgus**	2 (40)	61 (64)	0.362	0.94 (0.05–18.34)	0.965
**Idiopathic**	0 (0)	48 (50)	0.058	3.96 (1.27–12.39)	**0.018**
**Correction location**			0.616	1.58 (0.24–10.49)	0.635
Distal femur alone	1 (20)	40 (42)			
Proximal tibia alone	2 (40)	24 (25)			
Both sites	2 (40)	32 (33)			
**Preoperative location of the centre of the mechanical axis**^**b**^			1.000	0.57 (0.03–11.01)	0.711
2	2 (40)	45 (47)			
3	3 (60)	51 (53)			

^a^CI indicates confidence interval

^b^Preoperative location of the centre of the knee mechanical axis: zones 2, 3, -2 and − 3

Bold values are statistically significant, *P* < 0.05

Values are presented as the mean ± SD (range) or frequency (percentage)

## Discussion

In the present study, 94% (95/101 knees) of the genu angular deformities were completely corrected, and the mean deformity correction period was approximately 13 months. Non-idiopathic genu angular deformity was found to be an independent risk factor for deformity correction failure and complications. The femoral correction velocity was significantly faster than the tibial correction velocity. The femoral correction velocity decreased with age, and the tibial correction velocity was slower in genu varum and non-idiopathic genu angular deformities.

TH is a widely used surgery for treating coronal genu angular deformity because of its predictable results, reversible process and minimal invasiveness [1, 6, 7]. Despite the existing risk of hardware-related complications and premature physeal arrest when using eight-Plates, their incidences were relatively low in the TH procedure compared to the use of staples [2, 3, 5]. In our study, the mean age of the patients was 4.69 years old and ranged from 1 to 10 years old. In all, 94.06% (95/101) of the genu angular deformities were corrected to standard lower limb alignment (centre of the mechanical axis zone: -1 to 1). Kumar S et al [9] and Danino B et al [15] found that the deformities were successfully corrected in 196 out of 215 patients (91.2%, mean age: 9.5 years old) and 342 out of 372 physes (92%, mean age: 12.5 years old), respectively. Our research suggested that younger children with genu angular deformities could have good results when TH surgery is performed using eight-Plate implants.

The overall femoral correction velocity (1.28° per month) was significantly higher than the tibial correction velocity (0.83° per month) (*p* < 0.001). This is consistent with the assumption in a previous study [16] that the growth rate of the distal femoral growth plate (9 mm per year) was higher than that of the proximal tibial growth plate (6 mm per month). Danino B et al [4] published a study on a series of 967 physes in 537 patients with various aetiologies undergoing TH for genu deformities (mean age: 11.35 years old) and measured a 0.77°/month change in the mLDFA and a 0.79°/month change in the mMPTA. However, no significant difference was found between the correction rates of the femur and tibia in their study, which was different from the results of our study. Ballal MS et al [17] described a total of 25 children with various aetiologies (37 legs and 51 segments, mean age: 11.6 years old) of coronal plane deformities around the knee and measured a mean correction rate of 0.7° per month in the distal femur and of 0.5° per month in the proximal tibia. In our research, the mean age was younger, and the overall correction velocity was higher than that in the mentioned studies. Ballal MS et al [17] also found that the overall correction rate decreased with age, which was consistent with our study. Kulkarni RM et al [11] collected data on a total of 24 children with various aetiologies (40 legs and 63 physes, mean age: 5.25 years old) who were under the age of 10 and who underwent surgery for coronal plane deformities. The mean overall rate of correction was 1.53°/month (below 5 years of age − 1.67°/month, above 5 years of age − 1.39°/month), which was similar to the rate in our study. Danino B et al.’s research [4] found that younger patients with more than three years of growth potential have greater success with TH. All of these studies suggested that younger children might have higher correction rates and be good candidates for TH, but they also need short-interval routine follow-up more than older children to prevent overcorrection.

We found that the aetiology of genu angular deformity was an independent risk factor, and the odds of surgical failure increased by 2.47 times with non-idiopathic genu angular deformity (Table 3). Kang S et al [18] did not find a significant difference in the rate of satisfactory hemiepiphyseal stapling correction between patients with multiple hereditary exostoses (MHE) and those with idiopathic deformities. However, because the MHE group had a slower correction velocity, the authors of the study also thought that TH should be considered at an earlier age for these patients. Jain MJ et al [19], in their research on Blount disease combined with a systematic review of the literature, found that severe deformity, the use of a titanium tension band plate (TBP) and obesity were more likely to lead to surgical failure. However, none of these factors was present in our research, which did not involve patients with Blount disease. Yilmaz G et al [20] collected data on 29 patients (50 lower extremities, mean age: 10 years old) with different types of skeletal dysplasia who were treated for genu angular deformities at two centres. They found that TH surgery using an eight-Plate is effective in very young patients, which is important in skeletal dysplasia. However, 7 patients (24%, 7/29) did not benefit from the TH procedure, which suggested that the failure rate was relatively high in non-idiopathic patients. The results were similar with those in the present study. Therefore, we should inform the patient’s parents about the risk of operation failure preoperatively, especially in the treatment of non-idiopathic genu angular deformities.

The initial SDA was not related to the correction velocity in the present research. This result was consistent with Eltayeby HH et al.’s [21] research. Our study did not find a significant relationship between the percentage of screw length accounting for femoral or tibial physis width and the correction velocity. Therefore, we recommended that epiphyseal protection during implantation might be more important than intentionally pursuing a parallel or divergent screw angle.

Complications, such as screw loosening, screw breakage and wound infection, have been reported in the literature to be 3.4-4% [9, 22]. In our research, 4.95% (5/101 knees) of screw loosening complications occurred in patients with non-idiopathic deformities. In a recent retrospective multicentre study by Danino B et al. [15], no hardware failure was observed in 372 physes in 206 patients with idiopathic angular deformities of the knee joint. In addition, we did not find a relationship between screw length and screw loosening or correction velocity. Therefore, we should also inform the patient’s parents about the possibility of implant failure, such as screw loosening, in the treatment of non-idiopathic genu angular deformities.

The prevalence of rebound after eight-Plate removal in the present study (2.97%, 3/101 knees) was similar to that in Kumar’s systematic review (2.3%, 8/350 knees) [9]. Park SS et al. [12] suggested a predictive model for rebound in children with idiopathic genu valgum corrected by TH using staples; the model indicated that a lower rate of correction and a higher body mass index (BMI) might have protective effects against rebound. However, in other studies using eight-Plates, obesity, younger age and rapid correction velocities were thought to be risk factors for rebound [1]. We did not find influencing factors by univariate or multivariate analysis (Table 4). The low incidence of rebound in our research made it difficult to investigate the risk factors for rebound. In addition, we perform a slight overcorrection to prevent the rebound phenomenon, as it has been proposed by some reports that slight overcorrection is essential and effective in preventing rebound, especially for younger and obese children [7, 10, 12]. Therefore, we should inform patients’ parents about the probability of rebound and choose an optimal time for hardware removal.

In the present study, linear regression analysis showed that for every one degree change in SDA, the mMPTA and mLDFA change by 0.35 degrees. This result was consistent with that from Sweeney KR’s research [23]. Because of the correlation of change between the SDA and the mMPTA and mLDFA, the changes in mMPTA and mLDFA observed by full-length lower extremity standing anteroposterior radiography can be indirectly inferred from the change in SDA measured by local radiography of the knee. Therefore, frequent full-length lower extremity standing anteroposterior radiography might be replaced by local radiography of the knee during follow-up to reduce radiation. Further investigation is needed.

Several limitations to this study need to be addressed. First, the intrinsic limitations of a retrospective study in a single centre cannot be avoided completely. BMI is not routinely recorded in our hospital. Second, there is heterogeneity in the aetiology of the deformities. However, the present research focused on the population younger than 10 years of age, and the various aetiologies of the deformities were also investigated as risk factors for efficacy, complications and rebound in TH using an eight-Plate implant. Third, the limited number of patients included may weaken the strength of the results from the multivariate analyses, especially for the low incidence of rebound deformity. However, the present study has the largest sample of patients younger than 10 years of age with TH using an eight-Plate implant for the management of genu deformities. Finally, the mean follow-up time after eight-Plate implantation was 12.71 months (range: 12–24 months), which is relatively short for obtaining more precise results about rebound deformities. However, our centre is the regional orthopaedic centre for children, and we believe that these patients would return for care if they developed rebound deformities beyond the follow-up period. Future studies should extend the follow-up time after plate removal.

## Conclusions

TH using eight-Plates is an effective procedure with a low incidence of complications and rebound for genu angular deformity in patients younger than 10 years old. It should be considered when the deformity does not improve after conservative treatment. For children younger than 10 years of age with non-idiopathic deformities, their parents should be informed preoperatively of the possibility of correction failure or screw loosening after eight-Plate implantation.

## Data Availability

The datasets used and/or analysed in the current study are available from the corresponding author on reasonable request.

## Abbreviations

TH: Temporary hemiepiphysiodesis
CI: Confidence interval
mLDFA: Mechanical lateral distal femoral angle
mMPTA: Mechanical medial proximal tibial angle
SDA: Screw divergence angle
